# Oxidative Stress and Alcoholic Liver Disease

**Published:** 1997

**Authors:** Jose C. Fernández-Checa, Neil Kaplowitz, Anna Colell, Carmen García-Ruiz

**Affiliations:** Jose C. Fernández-Checa, Ph.D., is an investigator, Anna Colell is a doctoral student, and Carmen García-Ruiz, Ph.D., is a research associate at the Instituto Investigaciones Biomedicas, Consejo Superior Investigaciones Cientificas and Liver Unit, Hospital Clinic i Provincial, Universidad de Barcelona, Barcelona, Spain. Neil Kaplowitz, M.D., is a professor of medicine and chief of the Gastrointestinal Division at the Center for Liver Disease Research, University of Southern California School of Medicine, Los Angeles, CA

**Keywords:** ethanol metabolism, physiological stress, oxidation-reduction, pathogenesis, alcoholic liver disorder, glutathione, mitochondria, inflammation, literature review

## Abstract

Toxic substances generated during the metabolism of alcohol in the liver may contribute to the development of alcoholic liver disease. These substances include highly reactive molecules that can destroy vital cell components through a process called oxidation. Cells are protected against oxidation by the action of certain enzymes, vitamins, and other substances, known collectively as antioxidants. An imbalance between oxidants and antioxidants can lead to oxidative stress, characterized by escalating cell damage. Evidence suggests that the major energy-generating structures within cells (i.e., mitochondria) may be especially sensitive to oxidative stress, resulting in diminished energy production. Medications that reduce oxidative stress in mitochondria may ameliorate liver disease.

The metabolism of toxic substances that enter the body occurs largely in the liver. Among these toxic substances is alcohol, which is eventually broken down into simple end products for easy elimination. However, certain byproducts generated during alcohol metabolism may be more toxic than alcohol itself and may contribute to the development of alcoholic liver disease (ALD) (Ashak et al. 1991). These byproducts include oxygen-containing molecules that can destroy vital cell components through a chemical process called oxidation.

The first report implicating oxidative processes in the development of ALD was published in the early 1960’s by DiLuzio, who observed that alcohol administration promoted the oxidative breakdown of cell membranes (DiLuzio 1964; DiLuzio and Hartman 1967). DiLuzio further demonstrated that substances which inhibit oxidation (i.e., antioxidants) could prevent alcohol-induced membrane damage. From this early research arose the concept that an imbalance between oxidants and antioxidants can create oxidative stress, a state marked by continued production of oxidizing agents and escalating cell damage. This article explores alcohol metabolism and oxidative stress, the balance between oxidants and antioxidants, and the potential use of antioxidants to treat ALD.

## Oxidative Stress

In general terms, oxidation reactions are those that remove hydrogen from a substance or add oxygen to it (or both). Carefully regulated by enzymes, oxidation is a key step in many metabolic pathways that synthesize cell components, process metabolic waste, or burn food for fuel. However, some metabolic processes—such as the oxidation of alcohol—generate powerful oxidants that can contribute to oxidative stress (see sidebar, p. 323).

**Alcohol Metabolism**

Alcohol can be metabolized in various organs, including the stomach, small intestine, and brain. Quantitatively, however, most alcohol metabolism occurs in the liver, primarily through processes that involve oxidation (see figure) (Ashak et al. 1991; Lieber 1990). The discussion here focuses on two important pathways of oxidative alcohol metabolism: the alcohol dehydrogenase (ADH) pathway and the cytochrome P450 pathway.

***Alcohol Dehydrogenase Pathway***

Most ingested alcohol is initially oxidized in the liver by the enzyme ADH. This enzyme strips two hydrogen atoms from each alcohol molecule, thereby converting the alcohol to acetaldehyde. An enzyme called aldehyde dehydrogenase (ALDH) subsequently converts the acetaldehyde to acetate, also by removing hydrogen.

To facilitate these reactions, the hydrogen removed during oxidation must be transferred to a substance that can serve as a hydrogen acceptor. In the case of ADH and ALDH, this hydrogen acceptor is an enzyme “helper” (i.e., coenzyme) called nicotinamide adenine dinucleotide (NAD). Upon accepting hydrogen, NAD is said to become chemically “reduced” and is referred to as reduced NAD (or NADH). NADH, in turn, can relinquish its newly acquired hydrogen to other metabolic reactions, thereby becoming available once again as a hydrogen acceptor.

The regeneration of NAD from NADH occurs within mitochondria, structures within cells where most of the cell’s energy is generated through a process that promotes the formation of reactive oxygen species (ROS). The acetate formed by ALDH can serve as fuel for the cell. The ratio of oxidized to reduced coenzymes can significantly affect the cell’s metabolic balance.

**Figure:**
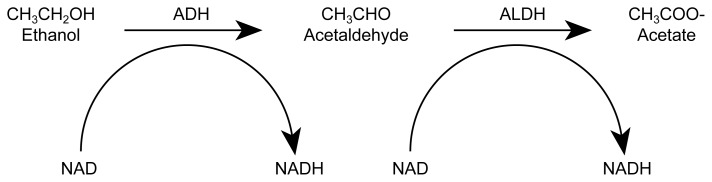
The metabolism of beverage alcohol (i.e., ethanol) by the alcohol dehydrogenase (ADH) pathway.

***Cytochrome P450 Pathway***

A secondary pathway of alcohol metabolism involves a group of detoxifying enzymes known collectively as cytochrome P450. Unlike ADH and ALDH, which are free in the water-soluble portion of the cell (i.e., the cytosol), the P450 complex is sequestered within tubular structures inside liver cells (Lieber 1987, 1990). Although these enzymes generally play a minor role in alcohol metabolism compared with ADH, long-term alcohol consumption can stimulate the P450 system to greater activity. The particular P450 component stimulated by alcohol is called CYP2E1. As with ADH, the immediate product of alcohol oxidation by CYP2E1 is acetaldehyde; the hydrogen acceptor in the reaction is a coenzyme similar to NAD.

CYP2E1 is metabolically active even in the absence of alcohol, forming small amounts of ROS (e.g., superoxide and hydrogen peroxide). Long-term alcohol exposure greatly increases ROS generation by cytochromes, which may lead to damage to the cell membrane (i.e., lipid peroxidation) and consequent cell damage.

—John J. Doria

*John J. Doria is a science editor of* Alcohol Health & Research World.

ReferencesAshakKGZimmermanHJRayMBAlcoholic liver disease: Pathologic, pathogenic and clinical aspectsAlcohol: Clinical and Experimental Research154566199110.1111/j.1530-0277.1991.tb00518.x2059245LieberCSMicrosomal alcohol oxidizing systemEnzyme3745511987310603110.1159/000469240LieberCSMechanism of alcohol induced hepatic injuryPharmacology and Therapeutics461171990218148610.1016/0163-7258(90)90032-w

Many of the oxidants generated by alcohol metabolism fall within a category known as reactive oxygen species (ROS’s). This article concentrates on two ROS’s: superoxide and hydrogen peroxide (see text box, p. 324). Trace amounts of these powerful oxidants are thought to help defend cells against invading bacteria and other microorganisms. When produced in excess quantities, however, superoxide and other ROS’s can injure cells by attacking vital cellular constituents, such as proteins, fatty substances (i.e., lipids) in cell membranes, or the molecules that make up the cell’s genetic material.

**Stable and Unstable Oxygen**

Oxygen atoms are an intrinsic part of most of the molecules that make up our bodies (see, for example, figure in sidebar, p. 323). A molecule of *atmospheric* oxygen is composed of two oxygen atoms bound together chemically (O_2_). This molecule is relatively stable, although inhaled oxygen does participate in metabolic reactions within the body’s cells. Reactive oxygen species are highly reactive, because they are chemically or electrically unstable. For example, superoxide (${\text{O}}_{2}^{-}$) is a highly reactive molecular fragment (i.e., a free radical) with a negative electric charge. Superoxide reacts with other molecules to become more stable and electrically neutral. Unlike water (H_2_O), which is stable, hydrogen peroxide (H_2_O_2_) has an extra hydrogen atom and is, therefore, unstable. (For the purpose of brevity, these descriptions are greatly simplified.)

### Alcohol Metabolism and the Cell’s Redox State

Hydrogen atoms that are removed during alcohol oxidation are transferred to a molecule called nicotinamide adenine dinucleotide (NAD) (see sidebar, p. 323). After receiving hydrogen, NAD is said to have been chemically “reduced” and is referred to as NADH. Thus, oxidation and reduction are complementary processes, because the oxidation of one substance entails the reduction of another. NAD–NADH shuttles hydrogen atoms back and forth between various oxidation-reduction reactions, helping to maintain an appropriate balance between oxidation and reduction in the cell (i.e., redox state).

Alcohol metabolism upsets the cell’s redox state, often by generating excess NADH. This glut of NADH inhibits other metabolic reactions that also might generate NADH. Some oxidation reactions that normally proceed by transferring hydrogen to NAD may continue by transferring hydrogen to oxygen instead. The reduction of oxygen under these conditions can produce ROS and lead to oxidative stress.

### The Inflammatory Response

Alcohol-induced oxidative stress contributes to the inflammation that accompanies severe ALD. White blood cells called neutrophils travel through the bloodstream to areas of liver cell damage, where they produce superoxide and release it into the surrounding tissue. This superoxide can form hydrogen peroxide and other types of ROS, which not only damage liver cells but attract additional neutrophils to the area.

**Figure:**
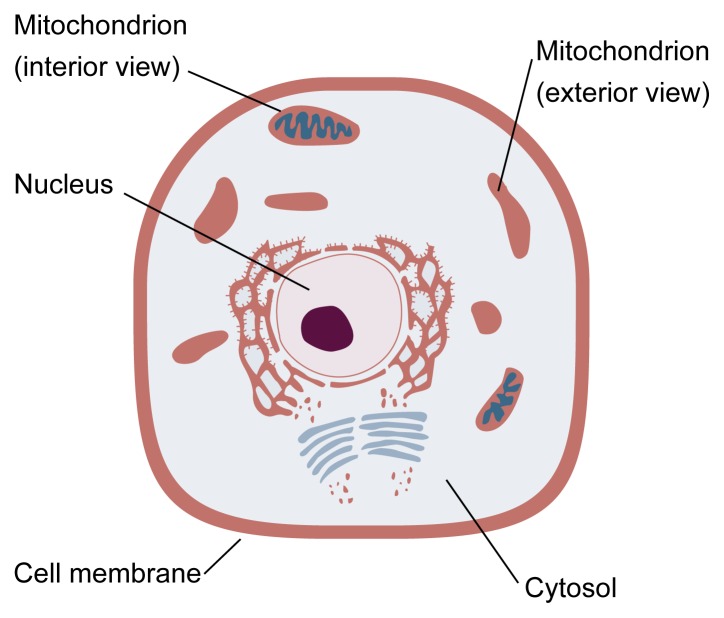
Schematic illustration of a typical cell. The nucleus contains the cell’s genetic “blueprint.” The remainder of the cell is filled with cytosol, a clear, water-soluble fluid. Specialized structures within the cytosol perform many of the cell’s functions. For example, mitochondria contain enzymes that generate most of the cell’s energy. A given cell may contain up to several thousand mitochondria. Each mitochondrion is surrounded by a two-layered membrane. The outer membrane controls the flow of substances into and out of the mitochondrion. The inner membrane is convoluted, providing extensive surface area for the attachment of enzymes. Enzymes

Liver injury and altered metabolism in ALD also may be generated by inflammation-promoting substances called cytokines (see article by McClain et al., pp. 317–320). For example, a cytokine called tumor necrosis factor-alpha (TNF-α) causes cell damage by promoting oxidative stress in mitochondria, the key energy-producing structures in the cell (see figure) (Fiers 1991; García-Ruiz et al. 1997). Thus, mitochondria may play a role in the development of ALD, particularly if antioxidant defenses are inadequate.

## Antioxidants

Cells are equipped with antioxidant strategies to squelch excessive oxidation. Among the cell’s antioxidant arsenal are specific enzymes, some of which work in sequential fashion. For example, some enzymes can convert superoxide to hydrogen peroxide, whereas other enzymes can convert hydrogen peroxide into water (Chance et al. 1979).

Additional antioxidants include vitamin E, vitamin C, and reduced glutathione (GSH). GSH, which participates in widely differing metabolic pathways, functions as an antioxidant through two distinct mechanisms. First, GSH can transfer hydrogens directly to oxidants without the intervention of an enzyme. Second, GSH can donate hydrogen atoms to assist an enzyme that coverts hydrogen peroxide to water (i.e., GSH-dependent peroxidase [GSH-Px]).

Just as an imbalance between oxidants and antioxidants can result in oxidative stress, an imbalance among antioxidants can promote oxidation. Such a situation could arise if antioxidants convert superoxide to hydrogen peroxide faster than the hydrogen peroxide can be converted to water. Hydrogen peroxide could then accumulate and participate in reactions that generate ROS (Fernández-Checa et al. 1997).

The situation just described is postulated to occur in mitochondria following long-term alcohol administration. This effect may result from an alcohol-induced depletion of mitochondrial GSH. The impairment of GSH-Px or limitation of its needed cofactor, GSH, leads to an accumulation of hydrogen peroxide in mitochondria, presumably generated by mitochondrial metabolism of superoxide.

## The Role of Mitochondrial GSH

The exact roles of antioxidant enzymes in human ALD are controversial. Nevertheless, research has demonstrated that an alcohol-induced depletion of mitochondrial GSH can contribute to cell damage by oxidative stress (Fernández-Checa et al. 1991, 1993; García-Ruiz et al. 1994).

Mitochondria cannot synthesize GSH. The high concentrations of GSH found in normal mitochondria are maintained by carrier proteins embedded in the membrane that surrounds each mitochondrion. These carriers selectively pump GSH from the fluid matrix of the cell (i.e., the cytosol) (see figure) into the mitochondrion. Alcohol appears to deplete mitochondrial GSH by disabling GSH carrier proteins.

Liver cells depleted of mitochondrial GSH become more susceptible to oxidative stress, as indicated by alterations of mitochondrial structure and function and a significant decrease in energy production. Mitochondrial GSH concentration can be returned to normal, however, using a synthetic GSH variant that can penetrate the mitochondrial membrane directly, bypassing the defective carrier. Cells treated with this modified GSH regain their resistance to oxidation, supporting a protective role for mitochondrial GSH (Fernández-Checa et al. 1993; García-Ruiz et al. 1994).

Mitochondrial GSH depletion can be partially prevented in alcohol-fed rats by the administration of S-adenosyl-l-methionine (SAM), a medication used to treat liver disease. Rats fed an alcohol-containing diet supplemented with SAM displayed a 40- to 50-percent increase in mitochondrial GSH compared with alcohol-fed rats not administered SAM (Colell et al. 1997). In addition, SAM treatment prevented the loss of mitochondrial function seen in alcohol-fed rats not administered SAM.

Additional studies suggest that the alcohol-induced depletion of mitochondrial GSH precedes the onset of ALD (Tsukamoto et al. 1985; 1986; Takeshi et al. 1992; Fernández-Checa et al. 1993). Thus, evidence indicates that depletion of mitochondrial GSH could contribute to the development of ALD by decreasing antioxidant capacity, thereby favoring the development of oxidative stress in mitochondria.

## Summary

Mitochondrial malfunction may play an important role in oxidative stress and the development of ALD. In the presence of oxidative stress, mitochondrial GSH depletion accentuates the adverse effects of ROS, increasing the susceptibility of liver cells to the oxidative effects of inflammatory cytokines. The molecular mechanisms whereby alcohol metabolism impairs the transport of GSH into mitochondria are under investigation. Studies of the mitochondrial GSH carrier at the molecular level may reveal the mechanism underlying the alcohol-induced impairment of GSH transport. Future studies are needed to establish the role of mitochondrial GSH depletion in ALD and to develop strategies to correct this defect.
